# Intra-Arterially Delivered Mesenchymal Stem Cells Are Not Detected in the Brain Parenchyma in an Alzheimer’s Disease Mouse Model

**DOI:** 10.1371/journal.pone.0155912

**Published:** 2016-05-20

**Authors:** Na Kyung Lee, Jehoon Yang, Eun Hyuk Chang, Sang Eon Park, Jeongmin Lee, Soo Jin Choi, Wonil Oh, Jong Wook Chang, Duk L. Na

**Affiliations:** 1 Department of Health Sciences and Technology, SAIHST, Sungkyunkwan University, Seoul, Republic of Korea; 2 Department of Neurology, Samsung Medical Center, Seoul, Republic of Korea; 3 Neuroscience Center, Samsung Medical Center, Seoul, Republic of Korea; 4 Stem Cell & Regenerative Medicine Institute, Samsung Medical Center, Seoul, Republic of Korea; 5 Laboratory Animal Research Center, Samsung Biomedical Research Institute, Gangnam-gu, Seoul, Republic of Korea; 6 Samsung Biomedical Research Institute, Samsung Advanced Institute of Technology (SAIT), Samsung Electronics Co., Ltd., Seoul, 135–710, Republic of Korea; 7 Biomedical Research Institute, MEDIPOST Co., Ltd., 463–400, Gyeonggi-do, Republic of Korea; Sungkyunkwan University, REPUBLIC OF KOREA

## Abstract

Mesenchymal stem cells (MSCs) have a promising role as a therapeutic agent for neurodegenerative diseases such as Alzheimer’s disease (AD). Prior studies suggested that intra-arterially administered MSCs are engrafted into the brain in stroke or traumatic brain injury (TBI) animal models. However, a controversial standpoint exists in terms of the integrity of the blood brain barrier (BBB) in transgenic AD mice. The primary goal of this study was to explore the feasibility of delivering human umbilical cord-blood derived mesenchymal stem cells (hUCB-MSCs) into the brains of non-transgenic WT (C3H/C57) and transgenic AD (APP/PS1) mice through the intra-arterial (IA) route. Through two experiments, mice were infused with hUCB-MSCs via the right internal carotid artery and were sacrificed at two different time points: 6 hours (experiment 1) or 5 minutes (experiment 2) after infusion. In both experiments, no cells were detected in the brain parenchyma while MSCs were detected in the cerebrovasculature in experiment 2. The results from this study highlight that intra-arterial delivery of MSCs is not the most favorable route to be implemented as a potential therapeutic approach for AD.

## Introduction

Alzheimer’s disease (AD) is the most common form of dementia and the number of individuals affected with AD is rising annually worldwide. Characterized as a degenerative disease, the symptoms are irreversible, and a definite cure is currently non-existent. Pharmaceutical companies have introduced various acetylcholinesterase inhibitors and NMDA receptor antagonists into the market as treatments for AD, however these drugs were not effective in altering the clinical course of AD patients [1].

Recently, emerging preclinical evidence has highlighted the therapeutic efficacies of stem cells, especially of mesenchymal stem cells (MSCs), in AD [2–6]. The optimized and accurate delivery of MSCs is a major determinant to the overall success of stem cell therapy. Through the completion of our phase I clinical trial, our team has confirmed the safety and tolerability of human umbilical cord blood-derived mesenchymal stem cells (hUCB-MSCs) that were delivered via intra-parenchymal administration in AD patients [7]. Although the safety and tolerability of MSCs have been affirmed, the small sample size precluded statistical analyses to determine the efficacy of MSCs in treating AD. Such results further underscore the importance of repeated administration and the delivery of stem cells through optimized administration routes.

At present, several routes exist to deliver stem cells or therapeutic agents to the brain including the intravenous and intra-parenchymal routes. The intravenous route is non-invasive but a critical drawback is that through systemic circulation a large proportion of the cells show high engraftment in the lungs [8–10]. On the other hand, intra-parenchymal administration allows focal engraftment of cells in the brain but requires surgical procedures [11, 12]. Out of the various administration routes, a particularly promising method of delivery is the intra-arterial (IA) route. IA administration bears clinical relevance because stem cells can be delivered directly to the brain, without passing the lungs through the first-pass metabolism, and wide-spread distribution is possible through the circle of Willis [13–16].

Although the IA route for the delivery of MSCs has been utilized in various animal models such as stroke and traumatic brain injury (TBI), the potential of this route has not been studied in transgenic AD models. As a result, the objective of this present study was to assess the feasibility of the intra-arterial (IA) route in successfully delivering human umbilical cord blood-derived MSCs (hUCB-MSCs) into the brains of non-transgenic WT (C3H/C57) and transgenic AD (APP/PS1) and to provide the grounds for clinical advancement of this delivery route for stem cell therapy of AD patients.

## Materials and Methods

### Ethical Statement

This experiment was approved by the Institutional Animal Care and Use Committee (IACUC) of the Samsung Biomedical Research Institute (SBRI) at Samsung Medical Center (SMC). The SBRI abides by the Institute of Laboratory Animal Resources (ILAR) guide and is also an accredited facility of the Association for Assessment and Accreditation of Laboratory Animal Care International (AAALAC International).

### Cell Culture and Preparation

Human umbilical cord blood-derived mesenchymal stem cells (hUCB-MSCs) were obtained from MEDIPOST Inc. (Biomedical Research Institute Co., Ltd, Gyeonggi-do, Republic of Korea) [17]. Passage 6 hUCB-MSCs were cultured in T75 flasks containing Minimum Essential Medium (MEM)α1x media (Gibco-Invitrogen, Carlsbad, CA, USA) supplemented with 10% fetal bovine serum (FBS; Biowest, Riverside, MO, USA) and 0.5% gentamicin (Thermo Fisher Scientific, Hudson, NH, USA) at 37°C, 5% CO_2_. When 80–90% confluency was reached, cells were washed with Dulbecco’s Phosphate Buffered Saline (DPBS; Biowest, Riverside, MO, USA) and were then trypsinized (0.25%, Trypsin-EDTA, Gibco-Invitrogen, Grand Island, NY, USA). Detached cells were re-suspended in phenol red free MEMα1x media prior to infusion.

### Animals

Double transgenic AD mice (APP/PS1) that express both a mouse/human amyloid precursor protein (Mo/HuAPP695swe) and a mutant human presenilin 1 (PS1-dE9) were purchased from Jackson Laboratories (stock number 004462; Bar Harbor, ME, USA). These transgenic mice were crossed with non-transgenic wild-type mice to generate offspring which were genotyped. This study consisted of a total of 24 mice: transgenic AD APP/PS1 (>12 months of age, n = 6) and non-transgenic wild-type C3H/C57 (n = 18) mice. The mice were maintained in a 12 hour light / 12 hour dark cycle and were fed *ad libitum*.

### Intra-arterial Delivery of hUCB-MSCs

**Fig 1:**
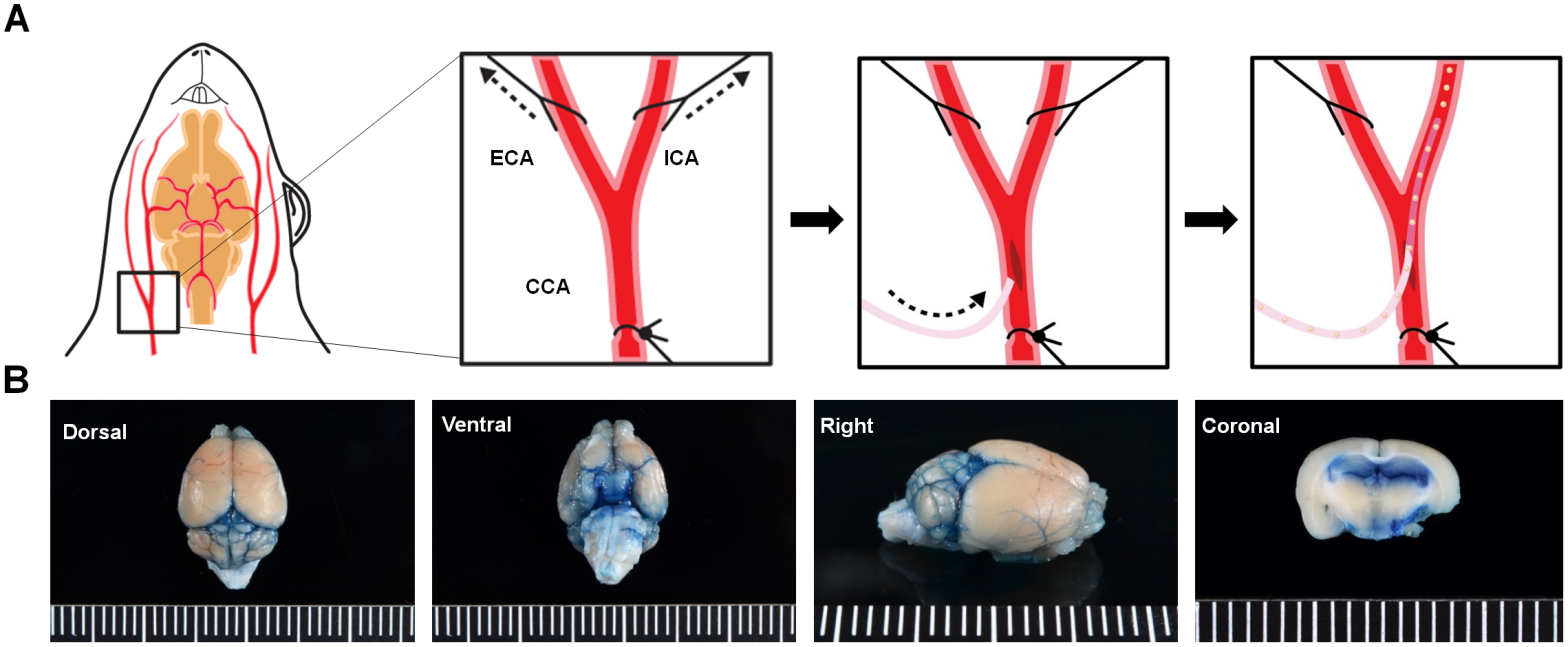
Surgical Procedure for Intra-arterial (IA) Administration. (A) The CCA, ECA, and ICA are exposed and a small incision is made on the CCA. The catheter is inserted and advanced towards the ICA and once stably positioned, the cells are manually injected. (B) Following infusion of the Evans blue dye (n = 12 WT mice), the dye was detected in various regions of the brain including the posterior communicating and middle cerebral arteries (MCA). CCA, common carotid artery; ECA, external carotid artery; ICA, internal carotid artery. Ruler Scale: Grid = 1 mm.

After initial anesthesia under 5% isoflurane (Hana Pharmaceutical Co., Ltd., Seoul, Republic of Korea), mice were placed in a supine position and were anesthetized under 2% isoflurane. A paramedian skin incision was made over the thyroid bone and the omohyoid and stemomastoid muscles were retracted so to expose the common carotid artery (CCA), internal carotid artery (ICA), and external carotid artery (ECA). A permanent ligature was made of the CCA using silk threads (6–0, Ethicon, Cincinnati, OH, USA) while the proximal segments of the ECA and ICA were temporarily ligated during the procedure (Fig 1A). An incision was made on the CCA using microscissors and a PE10 catheter line (Becton Dickinson, Franklin Lakes, NJ, USA) was inserted into the CCA and advanced towards the ICA. As a preliminary test to confirm the method of delivery, 0.2% Evans Blue (Sigma-Aldrich, St. Louis, MO, USA) was injected into the ICA of WT mice (n = 12) and were immediately sacrificed through decapitation to detect the staining of the arteries before wash-out (Fig 1B).

### Labeling and *in-vitro* Characterization of hUCB-MSCs Labeled with a Dual-modal Imaging Agent

MSCs that reached a confluency of 80–90% were treated with NEO-LIVE^™^-Magnoxide675, a dual-modal imaging agent [18, 19], at a concentration of 0.4 mg/mL according to the manufacturer’s instructions (Biterials, Seoul, Republic of Korea). NEO-LIVE labeled hUCB-MSCs were fixed in 4% paraformaldehyde (Biosesang, Gyeonggi-do, Republic of Korea) and mounted using a 4’,6-diamidino-2-phenylindole (DAPI) mounting medium (Vectashield, Vector Labs, Burlingame, USA) for observation under a confocal microscope (LSM700, Carl Zeiss AG, Jena, Germany). Labeled cells were also fixed in 1% paraformaldehyde and labeling efficiency was quantified using the fluorescence-activated cell sorting (FACS) Calibur instrument (Becton Dickinson, Franklin Lakes, NJ, USA). Previously reported methods [17] were used to induce osteo-, adipo-, and chondrogenic differentiation in unlabeled and NEO-LIVE labeled MSCs *in-vitro* and the following specific stains: Alkaline phosphatase, Von Kossa, Oil Red O, and immunohistochemical staining of collagenase II (Col II) were used to confirm each differentiation method. Cell viability was assessed by performing the 3-(4,5-dimethylthiazol-2-yl)-2,5-diphenyltetrazolium bromide (MTT; Sigma-Aldrich, St. Louis, MO, USA) assay as previously reported [20].

### Experiment 1: Infusion of Dual-modal Imaging Agent Labeled hUCB-MSCs through the IA route

**Fig 2:**
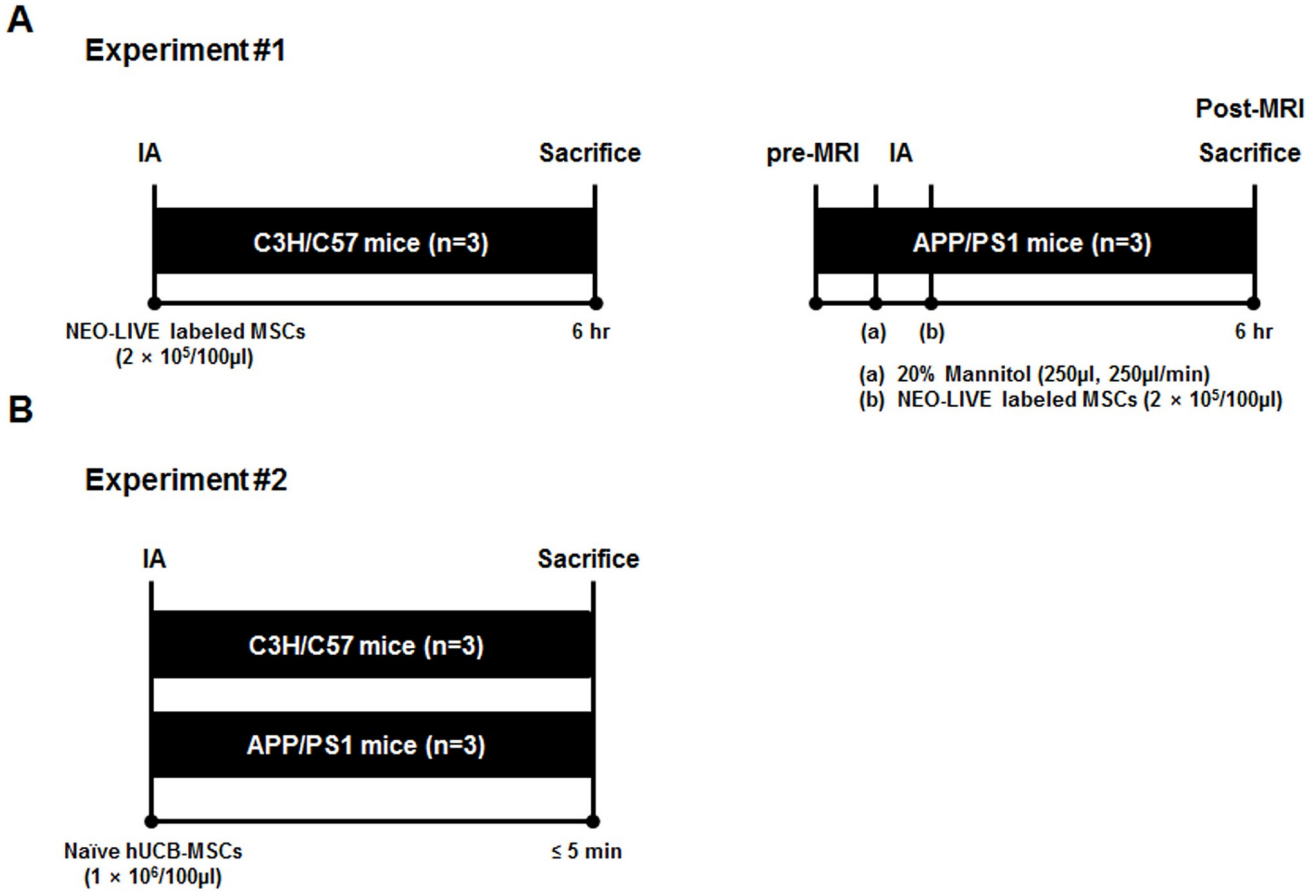
Schematic Illustration of the Overall Design of the Study. (A) Depiction of the injection procedure for the WT (n = 3) and APP/PS1 (n = 3) mice in experiment #1. (B) Illustration of the experimental design used for experiment #2 (n = 6).

A total of 6 mice were used for experiment 1. A schematic of experiment 1 is shown in Fig 2A. Non-transgenic WT (n = 3) and transgenic AD (n = 3) mice were infused with NEO-LIVE labeled hUCB-MSCs (2 × 10^5^/100μl) and then sacrificed through cardiac perfusion after 6 hours. To enhance the permeability of the BBB, the AD group received an initial infusion of 20% mannitol (250μl, 250μl/min; JW Pharmaceuticals, Seoul, Republic of Korea) using a micro-infusion pump (Harvard Apparatus, Holliston, MA, USA). MSCs were then administered within 5 minutes after mannitol infusion. This time gap in between mannitol and MSC infusion was used based on past reports [21]. Prior to being sacrificed, the AD group also underwent MRI to track the distribution of the cells real-time.

### Magnetic Resonance Imaging

Agarose phantoms were prepared by mixing 0.8% agarose (Bioplus, Gyeonggi-do, Republic of Korea) into tubes containing a varying number of NEO-LIVE labeled hUCB-MSCs. All phantom and *in-vivo* images were taken with a 7T/20 MRI System (Bruker-Biospin, Fällanden, Switzerland). Phantom MR images were acquired by using a multislice multiecho (MSME) spin echo sequence with the following parameters: Repetition Time (TR) / Echo Time (TE) = 2500 / 11–176 msec, slice thickness = 2 mm, number of averages = 1. MR images of the mouse brain were obtained by using a spin echo sequence and a quadrature coil. The following parameters were used: TR/TE = 2500 / 20 msec, slice thickness = 0.7 mm, number of averages = 8. Mice were anesthetized under 2% isoflurane while obtaining the MR images. A T2* gradient echo sequence was utilized to acquire MR images by using the following parameters: TR/TE = 303.805 / 18 msec, slice thickness = 0.7 mm, flip angle (FA) = 45°, number of averages = 8.

### Tissue Fixation and Prussian Blue Staining

At post 6 hours, WT and AD mice were sacrificed through cardiac perfusion and the brains were harvested and fixed in 4% paraformaldehyde for 24 hours. Sections (4μm) of the tissues were made using a microtome (Thermo Fisher Scientific, Hudson, NH, USA). Following de-paraffinization and rehydration in alcohol, tissue sections were treated with a working iron solution (equal mixtures of potassium ferrocyanide and hydrochloric acid) for 10 minutes (Sigma-Aldrich, St. Louis, MO, USA). Slides were then washed in distilled water (DW) and counterstained with Nuclear Fast Red for 5 minutes.

### Experiment 2: Infusion of Naïve hUCB-MSCs Through the IA Route

Using the surgical procedure described above, a total of 6 mice (non-transgenic WT: n = 3, transgenic AD: n = 3) were infused with naïve hUCB-MSCs (1 × 10^6^/100μl) and were sacrificed 5 minutes after the infusion (Fig 2B). The mice in this experiment did not receive a pre-infusion of mannitol and were not sacrificed through perfusion. Due to complications with the surgical procedure, MRI was not performed for the groups in experiment 2.

### Immunostaining

The following primary antibodies: anti-mitochondria antibody (1:200; Millipore, Billerica, MA, USA), CD31 (1:50; Abcam, Cambridge, MA, USA), and secondary antibodies: Alexa Fluor 488 conjugated goat anti-mouse, Cy3 conjugated goat anti- rabbit (1:250; Jackson ImmunoResearch Europe Ltd., Newmarket, UK), Dako EnVision + System-HRP Labelled Polymer anti-mouse (Dako, Carpinteria, CA, USA) were used for immunostaining.

After de-paraffinization and rehydration of the tissue in alcohol, heat induced antigen retrieval was performed using pH 6 1X citrate buffer (Dako, Carpinteria, CA, USA). Subsequently, slides were treated with hydrogen peroxide and protein serum (Dako, Carpinteria, CA, USA) for 10 and 20 minutes, respectively. For 3,3’-Diaminobenzidine (DAB) staining, tissue sections were stained with the primary antibody for an hour at room temperature, followed by staining with the secondary antibody for 30 minutes. After several washings, a DAB-substrate mixture (Dako, Carpinteria, CA, USA) was used for color development, and the slides were counterstained with Mayer’s hematoxylin (Dako, Carpinteria, CA, USA). In all immunofluorescence staining procedures, the primary antibody was incubated overnight at 4°C and samples were mounted in DAPI-containing mounting medium (Vectashield, Vector Labs, Burlingame, CA, USA). Stained slides were imaged using an inverted (U-HGLGPS, Olympus, Japan) or confocal microscope (LSM700, Carl Zeiss AG, Jena, Germany).

To create cell paraffin blocks, detached MSCs were suspended in egg albumin (Thermo Fisher Scientific, Hudson, NH, USA) and 95% ethanol. The white, solid precipitate that was produced from sonication and centrifugation was made into a paraffin block [22] and the sections from the block were used for staining.

### Statistical Analysis

All data are expressed as mean ± standard error of mean (SEM). A P value <0.05 was considered statistically significant. Differences between groups were examined using the Student t-test.

## Results

### Staining of Mouse Cerebral Blood Vessels by the IA Injected Evans Blue Dye

Utilizing the surgical procedure illustrated in Fig 1A, the Evans Blue dye was detected in the arteries of the mouse brain. From the basal view, arteries branching out from the internal carotid artery such as the posterior communicating and middle cerebral arteries (MCA) were stained with the dye (Fig 1B). Staining of the MCA was also observed in the cortex from the dorsal view. The branches of the MCA are known to penetrate into the inferior horn of the lateral ventricle. Successful staining of the lateral ventricles was observed upon coronal section of the brain while extravasation of the dye into the parenchyma was not observed (Fig 1B).

### Labeling of hUCB-MSCs with a Dual-Modal Imaging Agent

**Fig 3:**
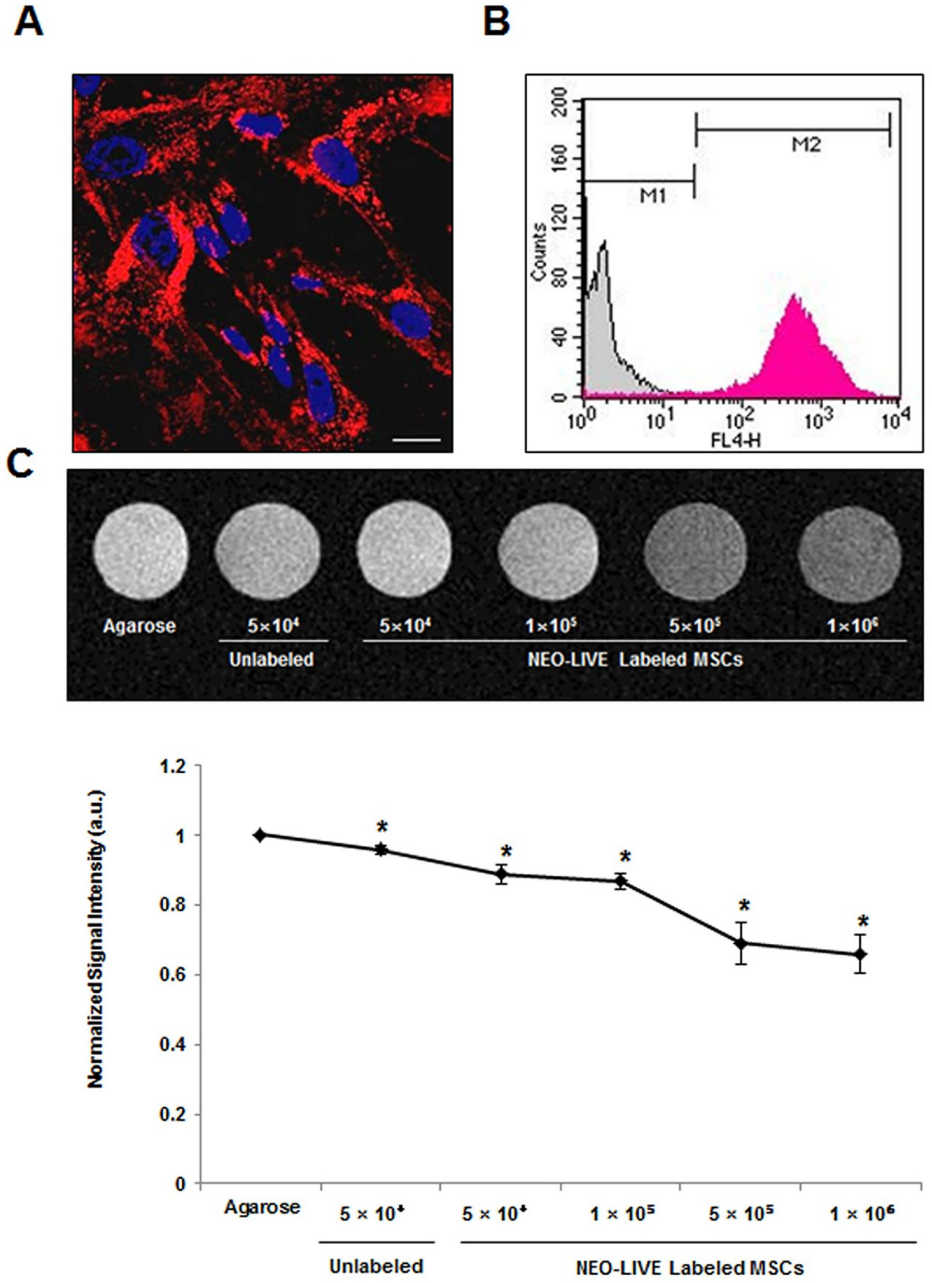
Characterization of hUCB-MSCs Labeled with a Dual-Modal Imaging Agent. (A) hUCB-MSCs were labeled with 0.4 mg/mL of NEO-LIVE for 24 hours. After the incubation period, cells were fixated with 4% paraformaldehyde and imaged using confocal microscopy. Internalization of NEO-LIVE nanoparticles (red) were evident in the cytoplasm (Scale bar = 20 μm). (B) The labeling efficiency of NEO-LIVE was also quantified through FACS. The labeling % was based on the average of 4 independent experiments (mean ± SEM, **P <0.01 compared with unlabeled control) (C) *in-vitro* agarose phantom MR images of NEO-LIVE labeled with hUCB-MSCs at varying cell doses. A reduction in signal intensity was observed with an increase in the number of labeled cells. The normalized signal intensity was calculated based on 3 independent experiments (mean ± SEM, *P < 0.05 compared with agarose control).

**Fig 4:**
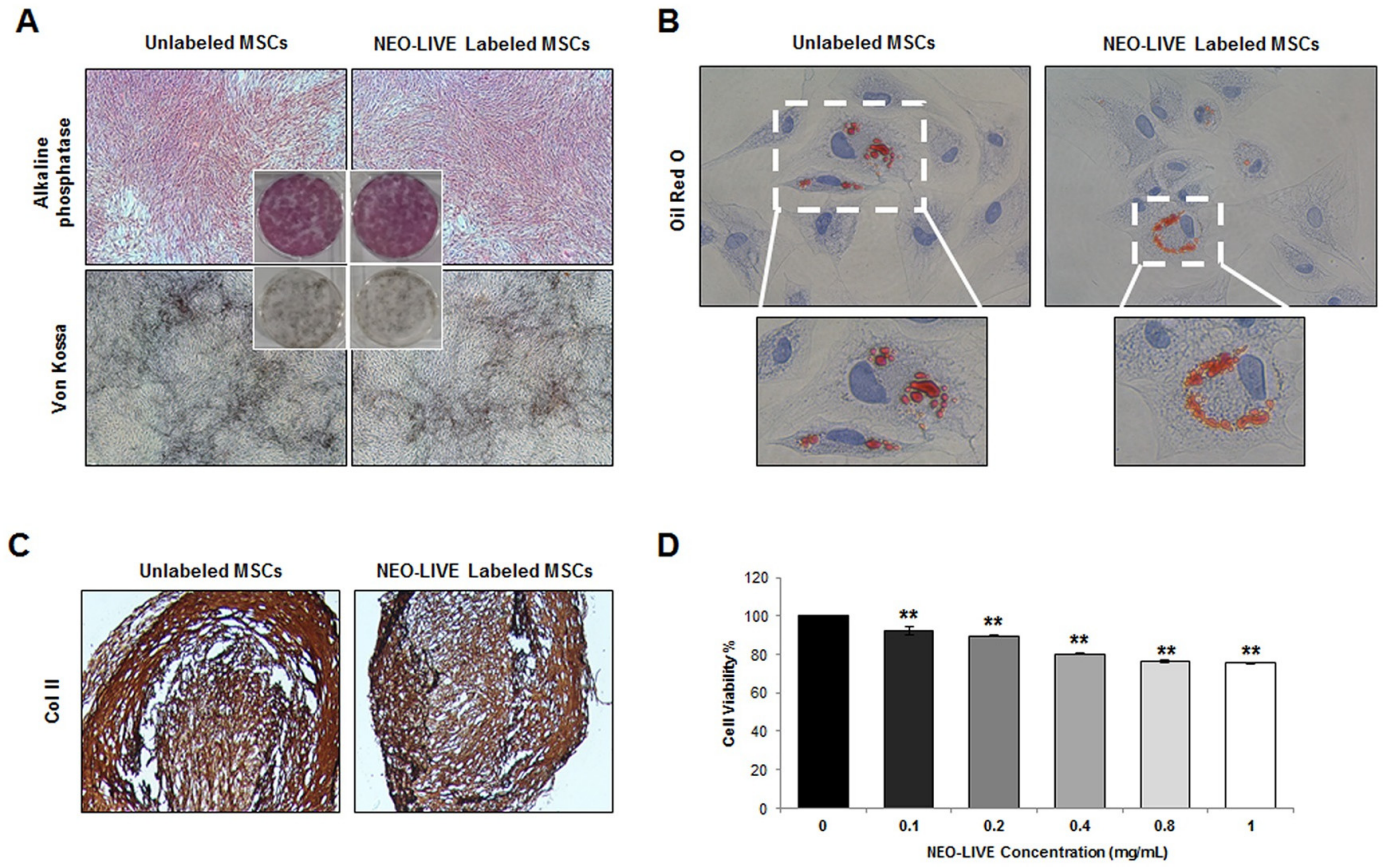
Maintenance of Multipotency and Viability of hUCB-MSCs Labeled with a Dual-Modal Imaging Agent. (A) Osteogenic (Alkaline phosphatase and Von Kossa), (B) adipogenic (Oil Red O), and (C) chondrogenic (immunohistochemical staining of collagenase II; Col II) differentiation of unlabeled and NEO-LIVE labeled hUCB-MSCs (40x, 400x, 100x magnification: A, B, C). (D) hUCB-MSCs were labeled with varying concentrations (0–1 mg/mL) of NEO-LIVE and the MTT Assay was used to measure cell viability. The cell viability percentages were calculated based on 6 independent experiments (mean ± SEM, **P < 0.01 compared with control).

The internalization of NEO-LIVE nanoparticles into the cytoplasm of the hUCB-MSCs was observed from the fluorescence images (Fig 3A) and the labeling efficiency measured by FACS was 97.1± 0.63% (Fig 3B). Based on the MR phantom images, a direct correlation was observed between the cell number and hypointensity (Fig 3C). Compared to the agarose sample, a gradual decrease in signal intensity was discernible with an increase in the number of labeled MSCs. Due to the sharp drop in signal intensity exhibited between 1 and 5 × 10^5^ cells, a dose of 2 × 10^5^ cells in experiment #1 seemed appropriate for detection in the MR images if engraftment in the brain was achieved. NEO-LIVE labeling did not have a lethal effect on the multipotency of hUCB-MSCs. Compared to the unlabeled MSCs, alterations in osteo-, adipo-, and chondrogenic differentiation were not noted from MSCs labeled with the nanoparticle (Fig 4A–4C). Furthermore, based on the MTT assay, the viability of hUCB-MSCs labeled with 0.4 mg/mL of NEO-LIVE was 80.1±0.42% (Fig 4D).

### Absence of Dual-Modal Imaging Agent Labeled hUCB-MSCs in the Mouse Brain Parenchyma

**Fig 5:**
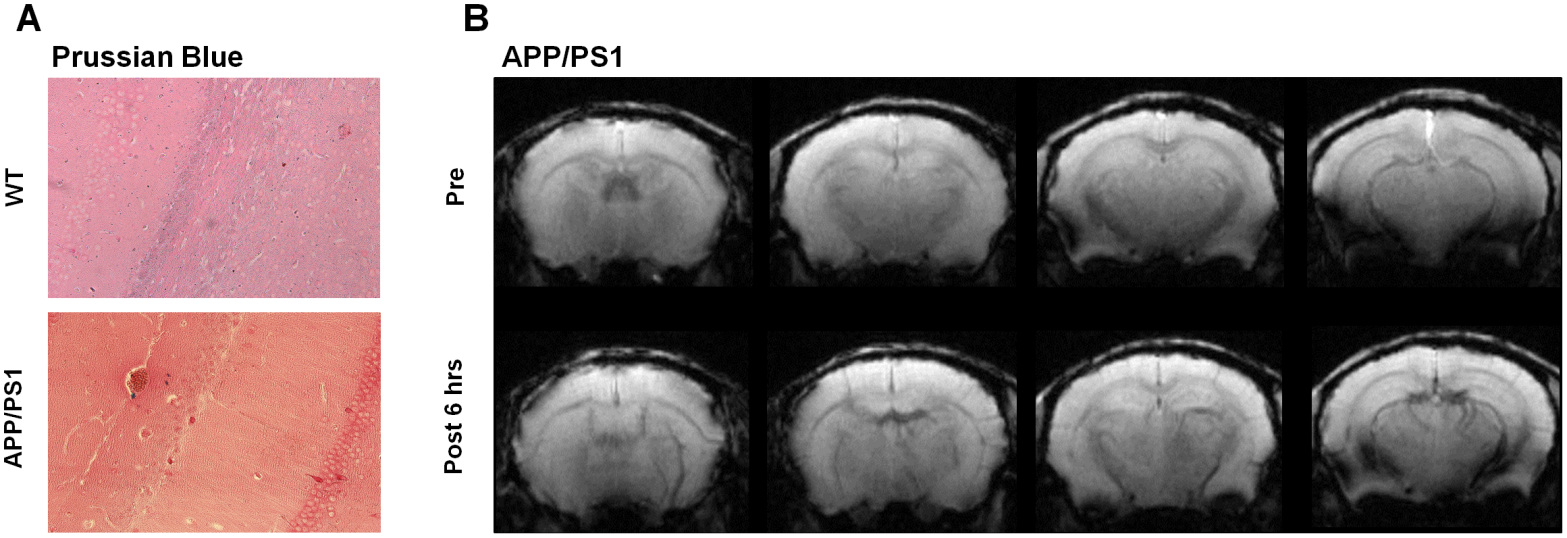
Absence of hUCB-MSCs in the Parenchyma in Mice Sacrificed After 6 Hours. (A) Prussian Blue stains of WT and AD brain sections (200x magnification). Iron positive, blue regions were not detected in the parenchyma. (B) Pre and post 6 hour MR images (7T, Bruker) acquired of APP/PS1 mice that were infused with 20% mannitol prior to NEO-LIVE labeled hUCB-MSC administration. Compared to the pre-image, no remarkable differences were noted except for a slight decrease in signal of the vessels.

Based on Prussian Blue staining, the presence of NEO-LIVE labeled hUCB-MSCs could not be detected in the parenchyma in any of the WT or AD mice brain sections (Fig 5A and 5B). A few iron-positive areas were discernible near vessel regions in AD mice (3 of 3 mice) that received a pre-infusion of mannitol but the total number of such areas was minimal (Fig 5A). Additionally, compared to the pre-images acquired prior to infusion, no prominent signals were detected in the parenchyma of the AD mice, although a slight decrease in the hypointensity of the vessels was observed in the MR images acquired at post 6 hours (Fig 5B).

### Detection of Naïve hUCB-MSCs in the Mouse Brain Cerebrovasculature

**Fig 6:**
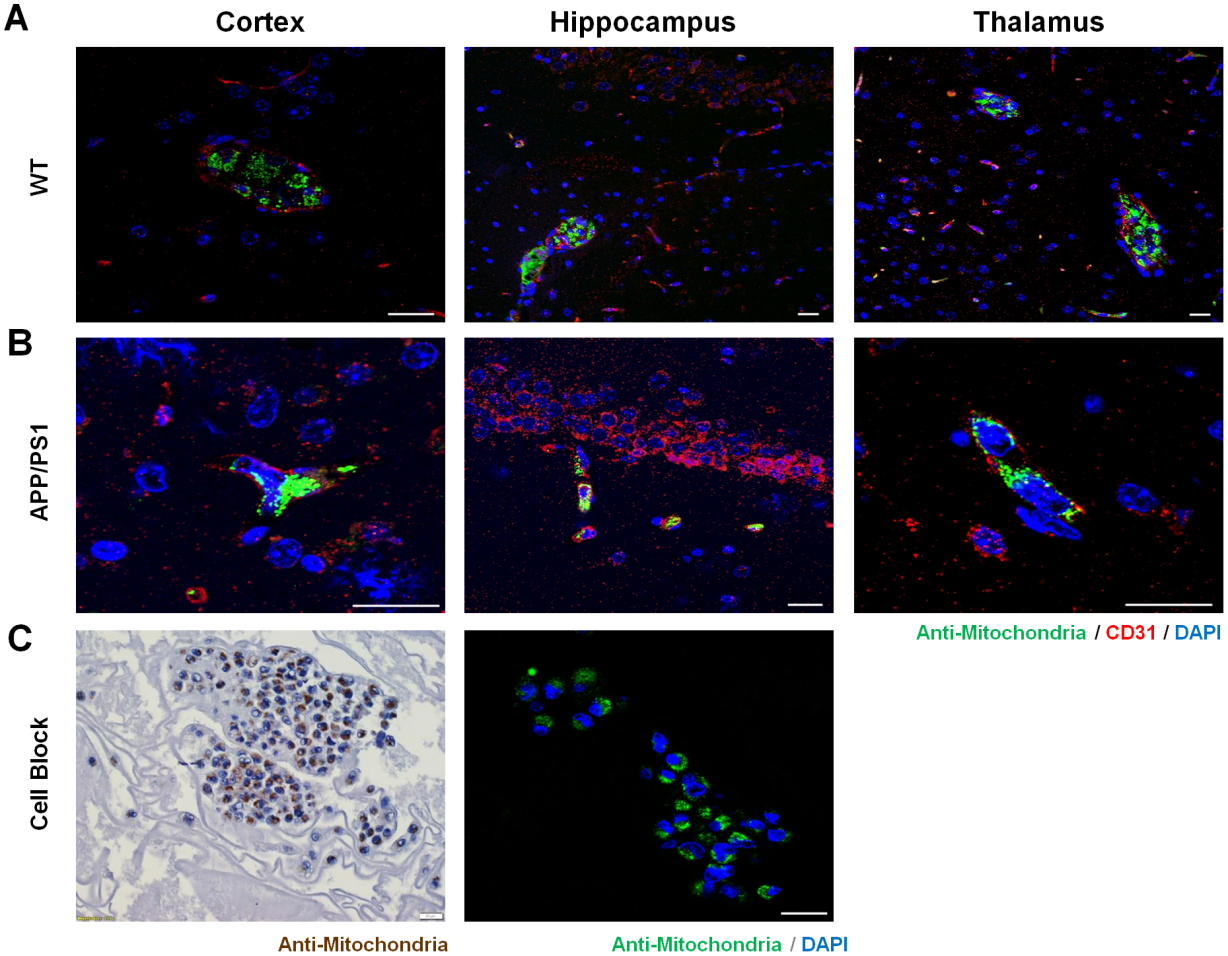
Localization of hUCB-MSCs in the Cerebrovasculature in Mice Sacrificed Within 5 Minutes. (A) The presence of human MSCs in the cerebrovasculature of WT and (B) APP/PS1 mice was observed by double staining the vessels using CD31 and the human cells using the anti-mitochondria antibody. (C) Suspended MSCs encapsulated by egg albumin were stained with the anti-mitochondria antibody through DAB and immunofluorescence staining. The morphology of the MSCs in the cerebrovasculature resembled that of the suspended MSCs fixated in paraffin blocks. Scale bar = 20 μm.

**Fig 7:**
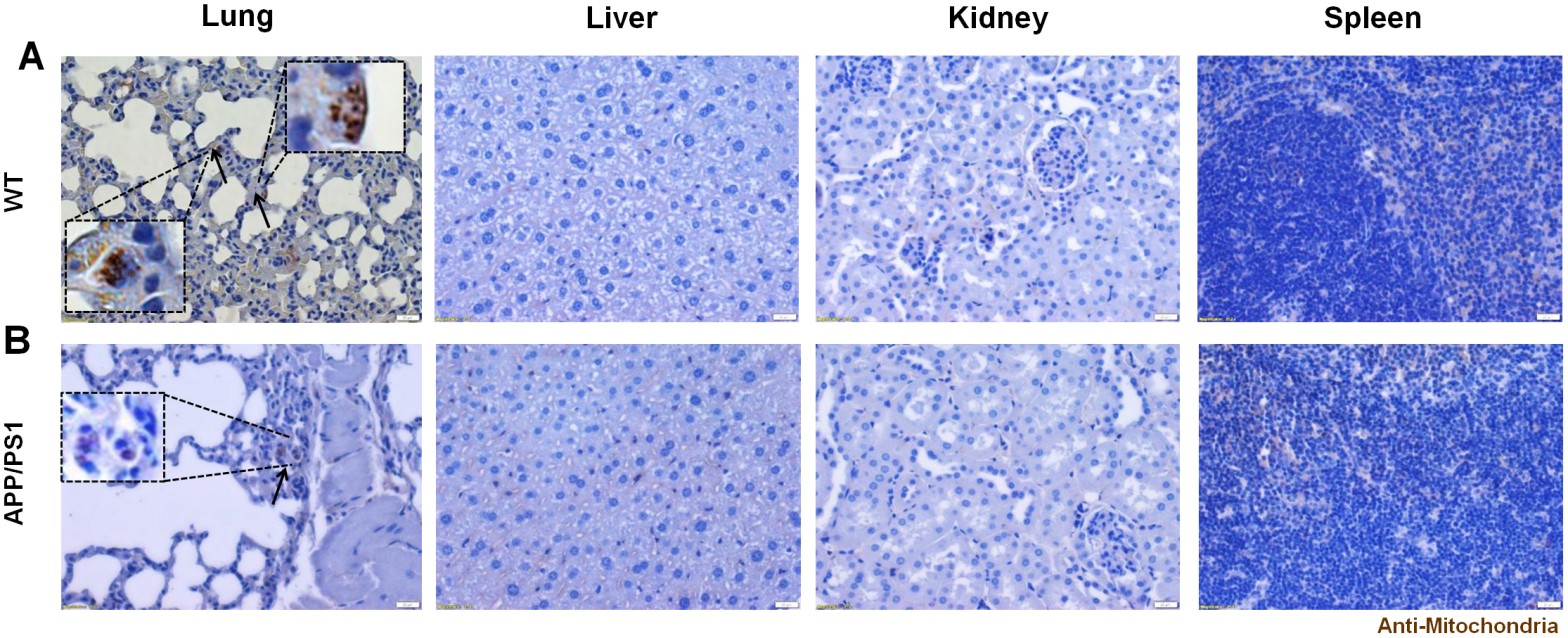
Distribution of hUCB-MSCs in Various Organs. Immunohistochemical staining (DAB) of human mitochondria in the lung, kidney, and spleen of (A) WT and (B) APP/PS1 mice that were harvested with 5 minutes following IA administration of naïve hUCB-MSCs. Several MSCs were seen in the alveolar walls of both WT and APP/PS1 mice while positive signals were not seen in the other organs. Scale bar = 20 μm. Solid arrows = human MSCs.

In both WT and AD mice, the engraftment of hUCB-MSCs in the brain parenchyma was not detectable, however the localization of MSCs was detected in the vessels of the brain (3 of 3 WT mice, and 3 of 3 AD mice) as confirmed by staining the vessels with CD31, a blood vessel marker, and the cells with the anti-mitochondria antibody (Fig 6A and 6B). The anti-mitochondria antibody staining pattern was observed as small specks that surrounded the nucleus of the cells in a halo-like form, which was equivalent to that observed in immunostaining of the cell paraffin blocks (Fig 6C). Localization of MSCs in the vessels was observed in various regions of the brain such as the cortex, hippocampus, and thalamus for both WT (Fig 6A) and AD mice (Fig 6B). At certain sites, an aggregation or bundle of cells were visible in the vessels, while smaller numbers of cells or only single cells were detected at other sites. While the infused cells were visible in the cerebrovasculature, none were observed in the brain parenchyma, and a small number of MSCs were also detected in the lungs of both strains (3 of 3 WT mice, and 3 of 3 AD mice), particularly in the alveolar walls (Fig 7). The presence of human MSCs was not detectable in other organs such as the liver, kidney, and spleen (Fig 7).

## Discussion

To the best of our knowledge, this is the first paper investigating on the delivery of human umbilical cord blood-derived mesenchymal stem cells (hUCB-MSCs) into the brains of transgenic AD (APP/PS1) and non-transgenic WT (C3H/C57) mice following administration through the intra-arterial (IA) route. The findings of our study indicate that MSCs cannot be successfully delivered to the brains of WT and AD mice through the IA route. An uncompromised BBB in AD could be a possible explanation of these results. In the past, numerous studies have reported the successful intra-arterial delivery of mesenchymal stem cells into the brains of stroke and traumatic brain injury (TBI) mice models. BBB impairment has been reported in TBI, stroke, and even multiple system atrophy (MSA) [23–26], however the integrity of the BBB is a controversial issue in Alzheimer’s disease (AD) [27–29]. Our results are in agreement with studies proposing that the BBB is not impaired in AD. In experiment 1, no striking differences in parenchymal cell engraftment were observed between the AD group that received a pre-infusion of 20% mannitol and the WT group that did not. These data indicate that the BBB was intact so that mannitol, a hyperosmolar agent, could not serve as a facilitating agent to aid the entry of hUCB-MSCs into the brain parenchyma.

Besides the BBB, other factors that might have contributed to the unsuccessful delivery of MSCs into the mice brains are wash-out, circulation, and cell dose. Upon review of the current literature, the unsuccessful delivery of MSCs in experiment 1 may be partly associated with the fast circulation rate in rodents [30] as well as the rapid clearance of human origin cells from the circulation in rodent models [15, 31, 32]. Considering these two points, in experiment 2, perfusion was excluded and the mice (both WT and AD) were sacrificed within 5 minutes. Additionally, to reduce chances of wash-out, the cell dose was also increased. With such changes, hUCB-MSCs were detected in the cerebrovasculature of both WT and APP/PS1 mice when sacrificed within 5 minutes. Again, the preservation of BBB integrity may explain why the MSCs were observed in the vessels but not the parenchyma of both strains. Recent reports have also addressed the homing and stabilizing effects of MSCs on BBB permeability which can act as an additive factor in hindering the crossover of MSCs from the vessel to parenchyma [25, 33].

The length of time that MSCs will remain in the cerebrovasculature is also an important factor in their delivery. Permanent persistence of cells in the brain seems unlikely due to the rapid clearance of the cells following delivery [15, 32]. Another factor that might expedite the rapid elimination could be the immunological response generated by human MSCs [15]. Furthermore, several groups have also reported on the death of human MSCs, through necrosis and apoptosis, or phagocytosis as alternative explanations for their clearance [31, 34, 35]. Considering the transient stay of the MSCs in the brain vasculature, the possibility of MSCs in exerting a therapeutic effect upon the disease environment through their paracrine activities is highly questionable.

In summary, the results from this study demonstrate that the intra-arterial route may not be the optimal delivery route to achieve parenchymal engraftment in both transgenic and non-transgenic AD mice. Thus, within the realms of AD, IA may not be the most optimal route to achieve maximal delivery of MSCs to the brain. Depending on the particular disease model, the effects of intra-arterial delivery of MSCs vary and thus, further investigation is crucial to determine the suitability and efficacy of the IA route for the disease of interest.

## References

[1] Casey M, Antimisiavis D, O'Brien J. Drugs for Alzheimer's Disease: Are They Effective? P&T. 2010;35(4):208–11. 20498822 PMC2873716

[2] Lee HJ, Lee JK, Lee H, Shin JW, Carter JE, Sakamoto T, et al The therapeutic potential of human umbilical cord blood-derived mesenchymal stem cells in Alzheimer's disease. Neurosci Lett. 2010;481(1):30–5. 10.1016/j.neulet.2010.06.045 20600610

[3] Caplan AI, Correa D. The MSC: an injury drugstore. Cell Stem Cell. 2011;9(1):11–5. 10.1016/j.stem.2011.06.008 21726829 PMC3144500

[4] Kim DH, Lee D, Chang EH, Kim JH, Hwang JW, Kim JY, et al GDF-15 Secreted from Human Umbilical Cord Blood Mesenchymal Stem Cells Delivered Through the Cerebrospinal Fluid Promotes Hippocampal Neurogenesis and Synaptic Activity in an Alzheimer's Disease Model. Stem Cells Dev. 2015;24(20):2378–90. 10.1089/scd.2014.0487 26154268 PMC4598918

[5] Kim JY, Kim DH, Kim JH, Lee D, Jeon HB, Kwon SJ, et al Soluble intracellular adhesion molecule-1 secreted by human umbilical cord blood-derived mesenchymal stem cell reduces amyloid-beta plaques. Cell Death Differ. 2012;19(4):680–91. 10.1038/cdd.2011.140 22015609 PMC3307982

[6] Kim JY, Kim DH, Kim JH, Yang YS, Oh W, Lee EH, et al Umbilical cord blood mesenchymal stem cells protect amyloid-beta42 neurotoxicity via paracrine. World J Stem Cells. 2012;4(11):110–6. 10.4252/wjsc.v4.i11.110 23293711 PMC3536832

[7] Kim H, Seo S, Chang J, Lee J, Kim C, Chin J, et al Stereotactic brain injection of human umbilical cord blood mesenchymal stem cells in patients with Alzheimer’s disease dementia: A phase 1 clinical trial. Alzheimer's & Dementia: Translational Research & Clinical Interventions. 2015;1:95–102. 29854930 10.1016/j.trci.2015.06.007 PMC5975048

[8] Wagner B, Henschler R. Fate of intravenously injected mesenchymal stem cells and significance for clinical application. Adv Biochem Eng Biotechnol. 2013;130:19–37. 10.1007/10_2012_155 23334265

[9] Park SE, Lee NK, Lee J, Hwang JW, Choi SJ, Hwang H, et al Distribution of human umbilical cord blood-derived mesenchymal stem cells in the Alzheimer's disease transgenic mouse after a single intravenous injection. Neuroreport. 2016;27(4):235–41. 10.1097/WNR.0000000000000526 26752148

[10] Toyoshima A, Yasuhara T, Kameda M, Morimoto J, Takeuchi H, Wang F, et al Intra-Arterial Transplantation of Allogeneic Mesenchymal Stem Cells Mounts Neuroprotective Effects in a Transient Ischemic Stroke Model in Rats: Analyses of Therapeutic Time Window and Its Mechanisms. PLoS One. 2015;10(6):e0127302 10.1371/journal.pone.0127302 26075717 PMC4468176

[11] Kean TJ, Lin P, Caplan AI, Dennis JE. MSCs: Delivery Routes and Engraftment, Cell-Targeting Strategies, and Immune Modulation. Stem Cells Int. 2013;2013:732742 10.1155/2013/732742 24000286 PMC3755386

[12] Walczak P, Zhang J, Gilad AA, Kedziorek DA, Ruiz-Cabello J, Young RG, et al Dual-modality monitoring of targeted intraarterial delivery of mesenchymal stem cells after transient ischemia. Stroke. 2008;39(5):1569–74. 10.1161/STROKEAHA.107.502047 18323495 PMC2857730

[13] Guo L, Ge J, Wang S, Zhou Y, Wang X, Wu Y. A novel method for efficient delivery of stem cells to the ischemic brain. Stem Cell Res Ther. 2013;4(5):116 24405845 10.1186/scrt327 PMC3854714

[14] Kristen D, Gremaud P, Novak V, Olufsen M, Vernieres G, Zhao P. Blood Flow in the Circle of Willis: Modeling and Calibration. Multiscale Model Simul. 2008;7(2):888–909. 19043621 10.1137/07070231X PMC2587352

[15] Mitkari B, Kerkela E, Nystedt J, Korhonen M, Mikkonen V, Huhtala T, et al Intra-arterial infusion of human bone marrow-derived mesenchymal stem cells results in transient localization in the brain after cerebral ischemia in rats. Exp Neurol. 2013;239:158–62. 10.1016/j.expneurol.2012.09.018 23059455

[16] Yavagal DR, Lin B, Raval AP, Garza PS, Dong C, Zhao W, et al Efficacy and dose-dependent safety of intra-arterial delivery of mesenchymal stem cells in a rodent stroke model. PLoS One. 2014;9(5):e93735 10.1371/journal.pone.0093735 24807059 PMC4012944

[17] Yang SE, Ha CW, Jung M, Jin HJ, Lee M, Song H, et al Mesenchymal stem/progenitor cells developed in cultures from UC blood. Cytotherapy. 2004;6(5):476–86. 15512914 10.1080/14653240410005041

[18] Kim JS, Kim YH, Kim JH, Kang KW, Tae EL, Youn H, et al Development and in vivo imaging of a PET/MRI nanoprobe with enhanced NIR fluorescence by dye encapsulation. Nanomedicine (Lond). 2012;7(2):219–29. 22175235 10.2217/nnm.11.94

[19] Park KS, Tae J, Choi B, Kim YS, Moon C, Kim SH, et al Characterization, in vitro cytotoxicity assessment, and in vivo visualization of multimodal, RITC-labeled, silica-coated magnetic nanoparticles for labeling human cord blood-derived mesenchymal stem cells. Nanomedicine. 2010;6(2):263–76. 10.1016/j.nano.2009.07.005 19699324

[20] Mosmann T. Rapid colorimetric assay for cellular growth and survival: application to proliferation and cytotoxicity assays. J Immunol Methods. 1983;65(1–2):55–63. 6606682 10.1016/0022-1759(83)90303-4

[21] Cosolo WC, Martinello P, Louis WJ, Christophidis N. Blood-brain barrier disruption using mannitol: time course and electron microscopy studies. Am J Physiol. 1989;256(2 Pt 2):R443–7. 2492773 10.1152/ajpregu.1989.256.2.R443

[22] Yamamoto R, Noguchi S, Tatsuta M, Kasugai H, Okano Y, Wada A, et al New cell-block method by utilizing egg albumin for fine needle aspiration biopsy. The Journal of the Japanese Society of Clinical Cytology. 1985;24(2):150–6.

[23] Cui LL, Kerkela E, Bakreen A, Nitzsche F, Andrzejewska A, Nowakowski A, et al The cerebral embolism evoked by intra-arterial delivery of allogeneic bone marrow mesenchymal stem cells in rats is related to cell dose and infusion velocity. Stem Cell Res Ther. 2015;6:11 10.1186/scrt544 25971703 PMC4429328

[24] Okuma Y, Wang F, Toyoshima A, Kameda M, Hishikawa T, Tokunaga K, et al Mannitol enhances therapeutic effects of intra-arterial transplantation of mesenchymal stem cells into the brain after traumatic brain injury. Neurosci Lett. 2013;554:156–61. 10.1016/j.neulet.2013.08.058 24016413

[25] Lee PH, Lee JE, Kim HS, Song SK, Lee HS, Nam HS, et al A randomized trial of mesenchymal stem cells in multiple system atrophy. Ann Neurol. 2012;72(1):32–40. 10.1002/ana.23612 22829267

[26] Song SK, Lee SK, Lee JJ, Lee JE, Choi HS, Sohn YH, et al Blood-brain barrier impairment is functionally correlated with clinical severity in patients of multiple system atrophy. Neurobiol Aging. 2011;32(12):2183–9. 10.1016/j.neurobiolaging.2009.12.017 20149484

[27] Poduslo JF, Curran GL, Wengenack TM, Malester B, Duff K. Permeability of proteins at the blood-brain barrier in the normal adult mouse and double transgenic mouse model of Alzheimer's disease. Neurobiol Dis. 2001;8(4):555–67. 11493021 10.1006/nbdi.2001.0402

[28] Do TM, Alata W, Dodacki A, Traversy MT, Chacun H, Pradier L, et al Altered cerebral vascular volumes and solute transport at the blood-brain barriers of two transgenic mouse models of Alzheimer's disease. Neuropharmacology. 2014;81:311–7. 10.1016/j.neuropharm.2014.02.010 24631967

[29] Choi JJ, Wang S, Brown TR, Small SA, Duff KE, Konofagou EE. Noninvasive and transient blood-brain barrier opening in the hippocampus of Alzheimer's double transgenic mice using focused ultrasound. Ultrason Imaging. 2008;30(3):189–200. 19149463 10.1177/016173460803000304 PMC3919133

[30] Chhabra L, Goel N, Prajapat L, Spodick DH, Goyal S. Mouse heart rate in a human: diagnostic mystery of an extreme tachyarrhythmia. Indian Pacing Electrophysiol J. 2012;12(1):32–5. 22368381 10.1016/s0972-6292(16)30463-6 PMC3273956

[31] Khabbal J, Kerkela E, Mitkari B, Raki M, Nystedt J, Mikkonen V, et al Differential Clearance of Rat and Human Bone Marrow-Derived Mesenchymal Stem Cells From the Brain After Intra-arterial Infusion in Rats. Cell Transplant. 2015;24(5):819–28. 10.3727/096368914X679336 24593908

[32] Lin H, Xu R, Zhang Z, Chen L, Shi M, Wang FS. Implications of the immunoregulatory functions of mesenchymal stem cells in the treatment of human liver diseases. Cell Mol Immunol. 2011;8(1):19–22. 10.1038/cmi.2010.57 21200380 PMC4002992

[33] Park HJ, Shin JY, Kim HN, Oh SH, Song SK, Lee PH. Mesenchymal stem cells stabilize the blood-brain barrier through regulation of astrocytes. Stem Cell Res Ther. 2015;6:187 10.1186/s13287-015-0180-4 26420371 PMC4588687

[34] Brenneman M, Sharma S, Harting M, Strong R, Cox CS Jr, Aronowski J, et al Autologous bone marrow mononuclear cells enhance recovery after acute ischemic stroke in young and middle-aged rats. J Cereb Blood Flow Metab. 2010;30(1):140–9. 10.1038/jcbfm.2009.198 19773802 PMC2893568

[35] Rosado-de-Castro PH, Schmidt Fda R, Battistella V, Lopes de Souza SA, Gutfilen B, Goldenberg RC, et al Biodistribution of bone marrow mononuclear cells after intra-arterial or intravenous transplantation in subacute stroke patients. Regen Med. 2013;8(2):145–55. 10.2217/rme.13.2 23477395

